# An Ontology-Driven Approach for Integrating Intelligence to Manage Human and Ecological Health Risks in the Geospatial Sensor Web

**DOI:** 10.3390/s18113619

**Published:** 2018-10-25

**Authors:** Xiaoliang Meng, Feng Wang, Yichun Xie, Guoqiang Song, Shifa Ma, Shiyuan Hu, Junming Bai, Yiming Yang

**Affiliations:** 1 School of Remote Sensing and Information Engineering, Wuhan University, Wuhan 430079, China; xmeng@whu.edu.cn (X.M.); 2012302580025@whu.edu.cn (F.W.); baijunming@whu.edu.cn (J.B.); 2016302580033@whu.edu.cn (Y.Y.); 2 Engineering Research Center for Geo-Informatics and Digital Technology Authorized by National Administration of Surveying, Mapping and Geoinformation, Wuhan 430079, China; 3 Institute for Geospatial Research and Education, Eastern Michigan University, Ypsilanti, MI 48197, USA; 4 Hubei Environmental Monitoring Central Station, Wuhan 430079, China; guoqiangsong2018@163.com; 5 Land and Resources Technology Center of Guangdong Province, Guangzhou 510075, China; whuma@163.com; 6 School of Resource and Environmental Sciences, Wuhan University, Wuhan 430079, China; shiyuanhu@sina.com

**Keywords:** ecological public health, heterogeneous geospatial sensors, computational intelligence, semantic sensor web, software agents

## Abstract

Due to the rapid installation of a massive number of fixed and mobile sensors, monitoring machines are intentionally or unintentionally involved in the production of a large amount of geospatial data. Environmental sensors and related software applications are rapidly altering human lifestyles and even impacting ecological and human health. However, there are rarely specific geospatial sensor web (GSW) applications for certain ecological public health questions. In this paper, we propose an ontology-driven approach for integrating intelligence to manage human and ecological health risks in the GSW. We design a Human and Ecological health Risks Ontology (HERO) based on a semantic sensor network ontology template. We also illustrate a web-based prototype, the Human and Ecological Health Risk Management System (HaEHMS), which helps health experts and decision makers to estimate human and ecological health risks. We demonstrate this intelligent system through a case study of automatic prediction of air quality and related health risk.

## 1. Introduction

Ecological public health promotes the concept that health depends on successful co-existence of the natural world and social relationships [1]. This co-existence focuses on increasing people’s awareness of environmental change and the interaction between the biological world and material consumption. Human health ultimately depends on the health of ecosystems. Many environmental indicators are included in health and ecological models to analyze or simulate interactions between the nature environment and its impacts on human society. Environmental data sources cover a wide range of fields, such as biological, physical, chemical, etc. and they are increasingly retrieved from heterogeneous fixed and mobile sensors. It is critical to integrate computational intelligence, such as intelligent data analysis and data-driven decision-making, to solve the problems of human and ecological health risks. In the coming big-data era, the effective use of environmental data for monitoring and computing human and ecological health risks is facing many challenges. Open data standards and open software paradigms are key concepts to offer us the best approaches [2]. Reis et al. [3] have identified a paradigm shift dealing with how the integration of models and smart sensors can contribute to environmental and human health.

Sensors are deployed all over the world, ranging from high level remote sensing to low level camera videoing, from earth observation to smart home monitoring, from advanced industry 4.0 to daily healthcare [4], and from the U.S. Army to Chinese civil engineering. Sensors are installed either as a fixed device or a mobile one, or even a wearable device to fit specific purposes, e.g., weather station monitoring, aircraft detection, athlete training, etc. At present, most sensors and their uses are managed by regular local file or database management technology for the Internet of Things (IoT)—enabled connection, retrieval and management [5]. However, the effective management of heterogeneous sensors is facing many challenges, since heterogeneous sensor nodes on the www (World Wide Web) or IoT are not well integrated or interconnected.

Traditional platforms of sensor networks (e.g., IrisNet [6], Hourglass [7] and Global Sensor Networks [8]) focus on composing densely wired or wireless networks for collecting and distributing observed data [9]. The platforms barely offered interoperability within local networks. People began to realize that there was a lack of uniform operation for resource reallocation, resource sharing, and the standard representation of a large volume of sensor data. Therefore, a generalized or standard-based sensor administration schema is needed to overcome the challenge of the shortage of interoperability within the sensor networks. The Open Geospatial Consortium (OGC) Sensor Web Enablement (SWE) [10] has been developed as an open consensus process for the intelligent use of all types of network-connected sensors and sensor systems.

As geospatial research focuses on spatial and temporal analysis of complex geological processes, modeling complex nonlinear systems, and supporting decision making, real-time and near real-time data collection using the processing capability of Geospatial Sensor Web (GSW) is indispensable [11]. Data mining and computational modeling that are critical for exploring and explaining the complex sensor network data can be greatly facilitated through ontology-based modeling. The GSW is used to deliver and show the most valuable geospatial information to the most needed researchers and the public through a series of object-oriented intelligent services [12].

In order to solve “intelligent” tasks at low, middle and high levels of the sensor architecture, soft sensors [13] and computational intelligence are frequently built on fuzzy logic, artificial neural networks, evolutionary computing, learning theory, and the probabilistic method, as the tools to determine the best indicators for each driver and goal in a smart city computing model [14]. Moreover, researchers intend to use the sensor web for attaining these kinds of intelligence acquiring, representation, and sharing, which are widely considered in a semantic web area [15]. For example, Gray et al. [16] described a semantic sensor web architecture for integrating multiple heterogeneous datasets, including live and historical sensor data, databases, and map layers. The semantic web can structure and tag heterogeneous and ambiguous sensor data to make them machine-understandable. A good number of researchers have been very active in sematic web studies, and they have created many types of ontology languages. The semantic specification of sensors and reasoning were surveyed about sensors and components based on the Commonwealth Scientific and Industrial Research Organization (CSIRO) ontology [17]. The W3C Semantic Sensor Network Incubator group (the SSN-XG) reviewed and analyzed existing sensor-centric and observation-centric ontologies [18], and proposed the Semantic Sensor Network Ontology (SSNO), which used CSIRO Sensor Ontology as the starting point for development, in order to create a domain-independent and full model for sensing applications. The SSN-XG group gave examples and described uses of the SSNO in recent research projects [19]. Geoscientists attempted to use the SSNO for modeling geospatial data, in order to help with designing a geo-ontology pattern for semantic trajectories [20]. There were also some successful applications, such as the ontology plan mode for geospatial data quality characterization in the semantic sensor web [21], and the SSN-based ontology which is used for water quality administration to sustain the water quality category [22]. The InWaterSense [23] was proposed as a SSN-based ontology framework and expert system for water quality monitoring. The richness of the ontologies and the capabilities of large reasoning offer much promise in revealing the value of environment information.

There are several typical GSW prototypes that semantically handle sensor networks. GeoCENS [24] consists of five core components, including a sensor web service engine, sensor web browser, semantic layer service, online social network, and recommendation engine. GeoSensor [25] provides a sensor query, data retrieval, sensor control, and semantic map functions in a web-based 2D and 3D Geographic Information System (GIS) platform. Sensapp [26] supports the abstraction of sensor data and services to standardized OGC interfaces/services, semantic annotation of such interfaces, enhanced discovery and composition of services, and data visualization on maps and charts. Overall, these platforms support satellite, aerial, and in situ sensors and use standardized models and interfaces in the service chain and for integration with GIS. To achieve ontology-driven reasoning, a geo-event was decomposed into several simple tasks based on ontology [27]. Durbha et al. [28] also proposed standards based middleware and tools, which exploited the instance data in the ontology concepts to realize mapping between different applications. This kind of middleware consists of syntactic standardization of metadata through open-standards-based sensor web components, which enriches the syntactical terms by using semantics to conceptualize them through ontology-based modeling in GSW.

However, there is a lack of cloud computing architecture to support big-data analytics. Knowledge-based systems are proposed as the solution for big-data analytics, including the application of automatic mode mapping to handle data-oriented heterogeneity, as well as semantic reasoning and ontology extraction that are used for innovative processing. A semantic link network was developed as a base system named Knowle [29], to organize and mine massive online health data, and it shows a promising potential for building, designing and developing which shows the potential for it forming the basis of designing and developing big-data analytics-based innovation frameworks for health. Cloud computing and stores can be combined into big-data analytics to provide more effective operations. In order to leverage the abilities of service-oriented decision support systems, big-data and analytics are put into the cloud [30]. Some new cloud-based infrastructures are proposed to model and assess environmental health and risks. For example, a cloud-enabled Climate Analytics-as-a-Service is discussed to overcome the big-data challenges in the climate science domain [31]. Cloud-based frameworks and intelligent platforms for home-diagnosis service and disease assessment over big medical data are proposed [32,33], but there are rarely specific GSW applications for certain ecological public health questions.

In short, sensor information is associated with geospatial elements (e.g., location point, trajectory line and monitoring area). At present, there is a lack of unified manipulation and specification representation for geospatial sensing and computing in the big-data and cloud computing environments. There are few means to share ecological public health resources in the heterogeneous geographic space. The distribution and share of the resources are usually closely coupled with particular locations, applications, or apparatuses used. Therefore, adding intelligent semantics to IoT and deep-learning to sensor management becomes an overwhelming trend [34]. New enhanced integration methods are needed to provide semantic web solutions. This study proposed an ontology-driven approach for integrating intelligence to manage human and ecological health risks in the GSW. We design a Human and Ecological health Risks Ontology (HERO) based on the SSNO for managing environmental sensors and computational models. The paper is organized as follows: Section 2 gives a general view of the sensor web environment, which includes our initiative, Sensor Web Management Framework (SWMF), HERO and the Human and Ecological Health Risk Management System (HaEHMS) which is a cloud-based GSW platform. Section 3 introduces the applications and a case study of an air quality assessment that was referred to software agents in the platform. The experiment and evaluation of comparing the system estimation and manual estimation are also described. Finally, Section 4 gives the conclusions of the full paper and suggests the topics of our future work.

## 2. Sensor Web Environment

### 2.1. Sensor Web Management Framework

**Figure 1:**
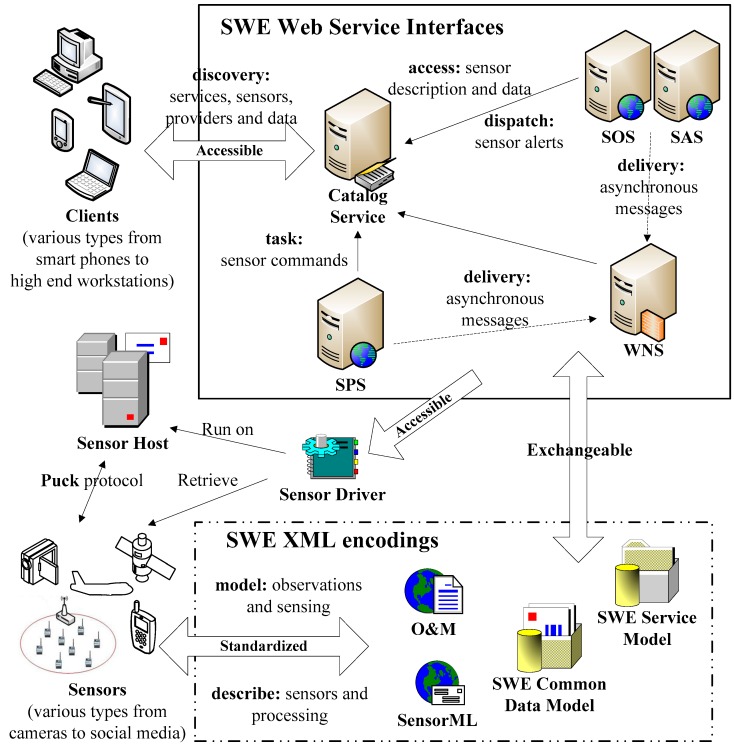
Components in the Sensor Web Management Framework.

Our initiative expands the OGC SWE by providing a standard set of common message models and service interface specifications for integrating network-connected sensors and sensor systems. The components in the Sensor Web Management Framework are illustrated in Figure 1.

The OGC Observations and Measurements (O&M) standard is used to encode and archive real-time observation results and values by sensors. OGC Sensor Model Language (SensorML) contains the Extensive Makeup Language (XML) schema and standard models for describing sensor systems and procedures. It also offers a list of the information that is needed to discover the sensors, the procedure of low level observations, and the process attributes. OGC SWE Common Data Model Encoding delimits the low level data models, which are used for exchanging referred data between nodes. The models are applied to construct, encode, and transmit data sets by self-description and semantic possibility. OGC SWE Service Model Implementation defines the type of data and interfaces that are common to services. The OGC Sensor Observation Service (SOS) and Sensor Alert Service (SAS) are web service interfaces that can be used to request, filter, and retrieve observations and sensor system messages. The OGC Sensor Planning Service (SPS) is applied to request client-driven gains and observations. The OGC Web Notification Service (WNS) is applied to the asynchronous delivery of information or early warnings of the SAS and SPS network services and other parts of the service work process. The Programmable Underwater Connector with Knowledge (PUCK) defines an agreement for the apparatus RS232 and web connections. PUCK transducers are installed and configured to solve the problem by defining an apparatus protocol to automatically store metadata and other information from the instrument itself. The SWMF also follows a Service Oriented Architecture (SOA) approach. A registry maintains a list of available sensors and queries using a group of web-based services. The Catalog Service contains dictionaries of phenomena, units of measure, sensor types and applications. This criterion of the SWMF includes formats for interfaces, agreements, and encodings so as to apply sensor data and services.

### 2.2. Semantic Sensor Web Management

It is hard to interconnect sensor webs and to realize efficient resource sharing and reuse because of their heterogeneity. As sensor networks become more and more common, the requirements of administering and inquiring the sensor web need to be aided by both standards and machine reasons. When massive numbers of sensors and amounts of data appear, it is very difficult for users to find the sensor and its related resources through the network. Even though the data is found, users cannot easily comprehend the acquired messages. Although the SWMF in Section 2.1 provides syntax interoperability, these criterions do not offer a semantic interoperability basis that can mitigate the inference of the advanced application development. The OGC SWE standard, among other things, helps the sensors to understand the process of measurement, the limitation of interoperability, the data switch due to XML, and the standardization of tags. However, they do not offer too much semantic interoperability, nor do they offer the foundation for reasoning or for obtaining intelligence.

**Figure 2:**
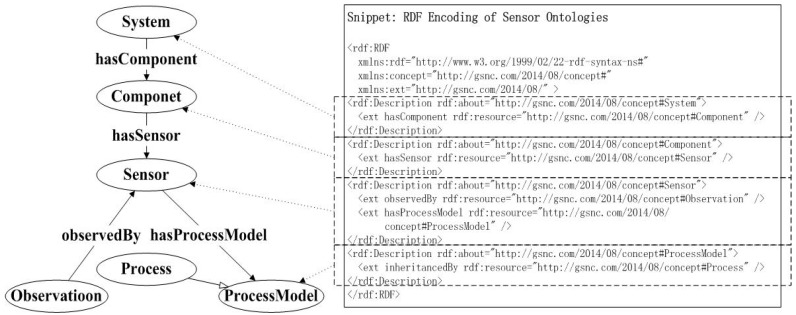
RDF/XML Encoding of Sensor Ontologies.

Ontologies and some other semantic technologies can become pivotal sensor web technologies, because they enhance the interoperability and integration of semantics, and because they promote the OGC standard to involve reasoning, classification and other types of assurance and automation. It is important to establish the core concepts and relationships of the sensor ontologies to describe the sensor knowledge. To solve this problem, the OGC SWE sensor ontologies frame could be organized into six core concepts: the systems, components, sensors, observations, process model and the process. It has been used as a general purpose method to conceptually describe or simulate the information that is executed in web resources that use various sorts of sentence structure markup and data serialization pattern. The abstract data matrix requires a specific syntax for representation and transmission, whereas the Resource Description Framework (RDF) has been given the XML syntax. Therefore, it carries on the advantages related to XML. Based on the kernel conception and the relations with the sensor ontologies, the RDF encoding is shown in part in Figure 2.

**Figure 3:**
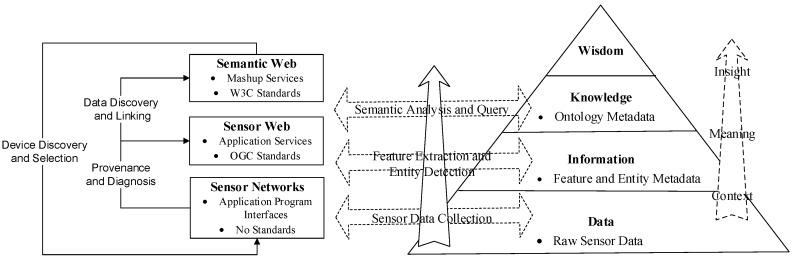
Sensor Data-to-Knowledge Pyramid.

Figure 3 shows a data-to-knowledge pyramid. The bottom section is the data layer without any processing and disposal (e.g., the air pollutant data observed by the transducers). It consists of multi-modal/level data collected from sensor and sensor networks; not only numerical data but multimedia data, such as binary pictures and streaming video. There are no ubiquitous standards for sensor applications in this layer. When we face the need to perform data fusion, we find that this layer lacks interoperability. The messages stratum is above the raw data stratum (e.g., associating the quality of the air with the site’s location). By extracting features from the raw phenomenological data and by detecting objects-events from features, and feature and entity metadata can be stored and queried in the form of annotations. Application services can manipulate the information through open standards (e.g., OGC standards). The knowledge stratum is applied to specify the semantic contents as well as to infer the association by making use of the ontology metadata. Semantic criterions will be integrated as data, and built into the linked data and mashups. Figure 3 also provides different perspectives on the categories, giving names for the three sets of use cases, which are (1) data discovery, (2) device discovery and selection, and (3) source and diagnosis. The reason for identifying multiple use case classification is that each use case demands distinct standards of detail to simulate the sensors, the observed functions, the environments and the conditions of use.

### 2.3. Human and Ecological Health Risks Ontology (HERO) Based on SSNO

**Figure 4:**
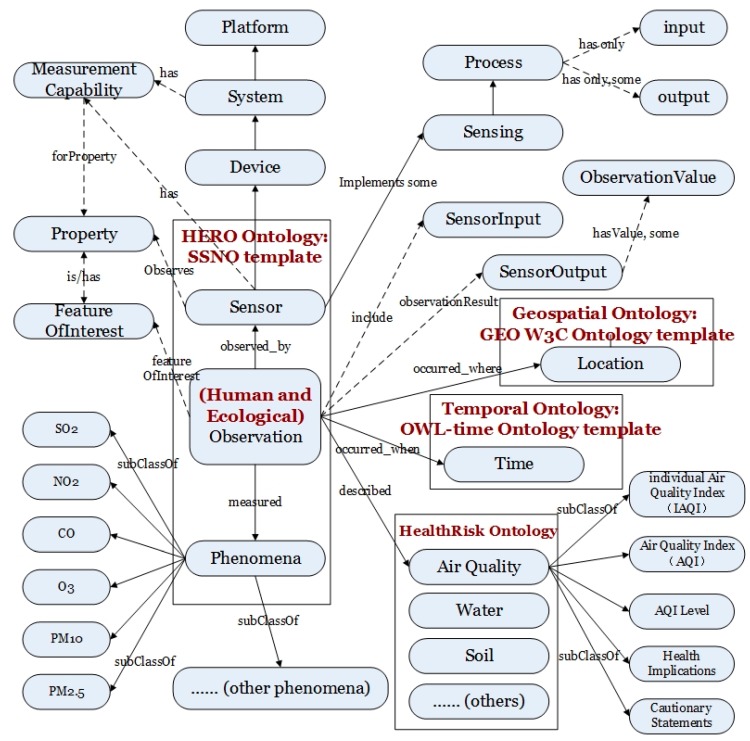
HERO Ontology.

Figure 4 shows a general picture of the categories and attributes in the ontology of the Human and Ecological health Risks Ontology (HERO), which is built based on the SSNO template. The ontology expression of the sensor connects with the field of what it gauges (the domain), the physical transducers (the grounding), and its abilities and computational procedures. Instead of attempting to describe each sensor and scene, the ontology exploits a general expression of the sensor and field characterization. It depends on high-level ontologies to define the field, and on an operation pattern that describes how the measurement is achieved.

The HERO includes Sensor, (human and ecological) Observation, and Phenomenon ontology components. The Observation can be described with the HealthRisk component to draw a picture of human and ecological health risks from the views of ambient air, surface water environment, soil environment, environmental noise, ecology, and biological environment, etc. The measured Phenomenon objects could be any natural element or event.

Take ambient air observation as an example; the analyzers for NO-NO_2_-NO_x_, SO_2_, CO, O_3_, and PM_10_/PM_2.5_ are described in the category of the Sensor component. The measurement capability of the Sensor is linked with the FeatureOfInterest of the Observation. The Location and Time components use the standard GEO W3C Ontology (http://www.w3.org/2003/01/geo/), that is, a simple RDF encoding for the WGS84 latitude and longitude values, and the OWL (Web Ontology Language)-time ontology as the templates. The geospatial and temporal ontology and the Observation Value component are connected with the Observation to describe the computational abilities. The Process component grants the capability of the heterogeneous sensors to compose chained sensing.

The intelligent agent for the geospatial and temporal processes will be shown in the Section 3.1.2. The case study of sensing and computing with the Sensor and Observation ontology will be illustrated in Section 3.2.

### 2.4. Human and Ecological Health Risk Management System (HaEHMS)

Government planners around the world must develop more efficient environmental information management systems to control human and ecological health risks, such as monitoring and evaluating the water quality of lakes, the soil structure of farmlands, the urban air quality of cities, and other environmental matters that are vital to people’s livelihoods, and also announcing the observation data to the public at a regular fixed period (always every day). In addition to the governmental agency, universities, research institutes, polluters, and citizens with green awareness are also able to acquire the environmental data by using their own standalone systems or citizen applications. In order to analyze the observations’ impacts on the ecosystems and human health, manage the human and ecological health risks, and deal with relevant decision-making issues, a web-based HaEHMS integrating the existing monitoring data, models, and processes was developed based on the SWMF and HERO.

**Figure 5:**
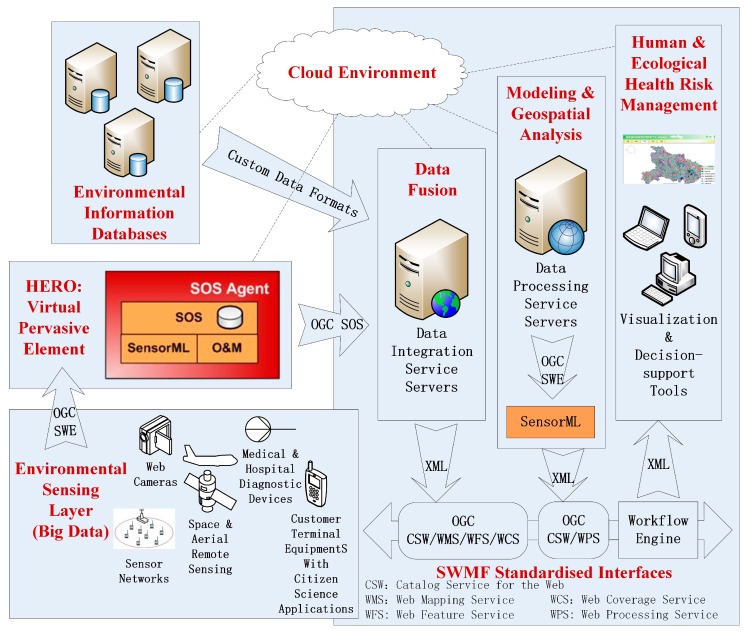
HaEHMS Technical Architecture.

Figure 5 shows the technical architecture of HaEHMS, which works in a cloud service environment. Various health big-data and environmental observations in the sensing layer will be computed with the HERO, which works as a virtual pervasive element in the form of a pre-defined SOS computational agent. Other organizations’ environmental information databases can be integrated into the data integration service server. Models and geospatial analysis can be also described as atomic processes. Through SWMF standardized interfaces and workflow engines, atomic processes could be orchestrated to achieve visualization and decision making for the human and ecological health risk management.

In addition to SWMF standardized interfaces and HERO virtual pervasive elements, HaEHMS consists of the following main subsystems:

(1) Environmental Sensing Subsystem

This is the observation and monitoring of subsystems that are supplying remotely sensed observations (e.g., images), in situ measurements (e.g., PM_2.5_ concentrations), civil findings (e.g., questionnaire surveys), and their transmission from regional and national backbone platforms. In situ apparatuses consist of transducers and other numerators of the monitoring Web that lie in and keep in touch with the phenomena where they are surveying. The main objective is to establish, operate, and to maintain a continuous time-series monitoring platform that is capable of tracking physical, chemical, and biological features. Another objective is to link this observatory with models, to better describe ecosystem changes and health risks, in order to provide current condition, short-term forecast, long-term projections, and trends analysis. Particularly, customer terminal equipment with citizen science applications can provide sustainable environmental sensing.

(2) Environmental Information Databases

The databases are used for information technology infrastructure and shared standards, protocols that are able to deliver real-time, delayed-patterns, and historical data for physical, chemical, and biological observations, and model-generated outputs. The sustainable environmental sensing data could be stored in databases. In particular, crowdsourcing information can be always treated as dynamic and remote sustainable environmental sensing information.

(3) Cloud Computing

Data provisioning systems are used to capture messages from the material world, interact with heterogeneous equipment and observation circumstances, possess high-speed processing abilities, and save and administrate massive data. In order to deal with the big sustainable environmental sensing data and related models, our application was enabled by the Amazon EC2 cloud hosting environment for data services. The cloud service is intended for web and mobile application data processing with analysis algorithms. Table 1 is the summary of resources and open sources used for implementation. At the Amazon Web Services (AWS) management console, an Amazon EC2 instance was first created. In the cloud server, the Amazon EC2 was used for a cloud computing environment to generate large instances, being operated in the Amazon machine image (AMI) on Linux. The Amazon S3 was a cloud storage environment for the processed results. The integrated development environments (IDEs) are also listed.

## 3. Application

The semantic web annotates data with geospatial, temporal, and topic semantic metadata. Throughout the SWMF, it is crucial to add semantic annotation to current criterion languages to promote the significance of the sensor observations. The HERO enhances its implication through adding semantic notes to SWMF’s existing criterion language. These notes offer more significant characterization and improve access to the data, and they play the role of a connection mechanism to make up the disparity between the principally syntactical XML-based metadata criterions of the sensor network, and the RDF/OWL-based metadata criterions. The HaEHMS is assembled by semantic web technologies through adding semantic notes, and constructing ontologies for interoperability, analysis, and reasoning over the heterogeneous multimodal data. This section mainly discusses the agent components and it gives a case study of querying the Air Quality Index (AQI) levels around the individual from the shared the Keyhole Markup Language (KML) sensor files that are annotated with RDF serializations in the HaEHMS.

### 3.1. Agent Components

The “agent components” in our study are the software programs that provided access to sensor data through web pages, and then trusted individuals could freely search, obtain, query and publish the data.

**Figure 6:**
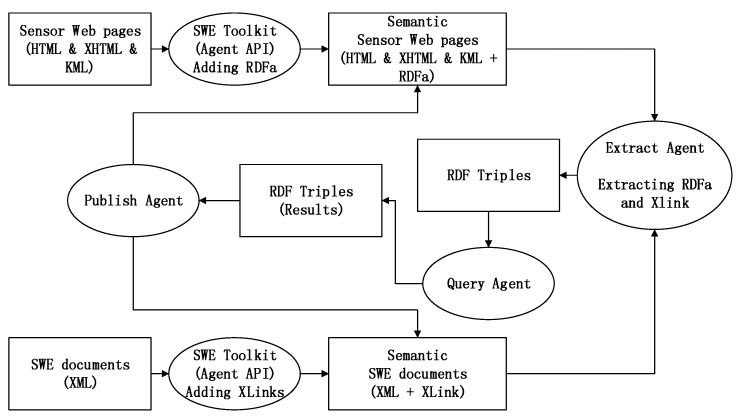
Semantic Enablement Agents.

Sensor knowledge was intended to have the characteristics of being open and free, and the semantic specification offers a powerful ability of sharing. Sharing is not restricted to standing up a SPARQL (the standardized query language for RDF) endpoint or to storing a static RDF document on the net server. In order to sustain the hybrid standards of KML, we developed the agent components, as shown in Figure 6, to realize the solution of admixing XLink for the XML detail, RDFa for the HTML, and the XHTML detail. In the solution, the interim from XML-based services to the RDF-based services is known as the lifting operation; as a comparison, we call the operation from the RDF to the XML the lowering operation. Software agents are developed for extracting, querying, and publishing XLink and RDFa automatically.

#### 3.1.1. Extracting Agent

The extracting agent works like a web crawler. Since much sensor data are hidden in the heterogeneous geospatial sensor web, we must find ways to export the data. We are developing two extracting software agents for web mixtures that are applicable at distinct levels of the multi-layered plan, including the inclusion of remote RDF (or OWL) resources in XML using XLink, or in HTML/XHTML/KML using RDFa. The Extracting Agents are developed, based on the technology for processing XML/XHTML microformats as conventional RDF syntaxes, through having each point of file, directly or indirectly, to a transformation to an RDF figure. RDFa allows a single parser to work for data from any area, and they offer a direct relationship between the RDF data and the XHTML/HTML file construction.

**Figure 7:**
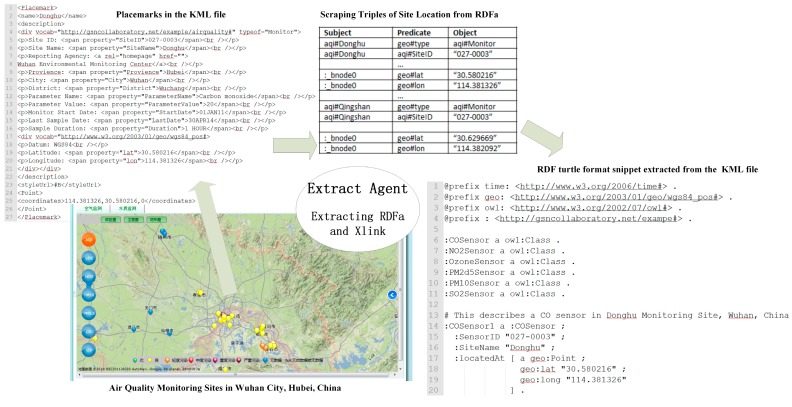
Extract Agent.

The RDF of the KML place mark is extracted by the agents, resulting in triples. The Table in Figure 7 shows the Scraping Triples of the Site Location from the RDFa. The RDF turtle format snippet extracted from the KML files could be used for reasoning.

The links in Figure 7 specify the inclusion of remotely managed sensor resources. The link may represent a mechanism for extending accessible substances through any kind of resource, using messages originating from remotely administrated substances, and the possibility between two documents of the same type, or between different types of documents. Semantic notes define how service abilities are mapped to semantic circumscription, to enable the findings or components of net services. The addition of semantic annotation (or say “markup”) to the existing SWMF, so as to offer semantic characterizations and to promote data standards being obtained, can provide more meaning to the characterizations, and can promote the significance of the sensing data. Adding semantic annotations is an ontology-based and useful way to model and share sensor knowledge, and it promotes viable utilizations. Appending semantic messages to former networks can improve the web page’s status from being human-readable to being machine-readable, and it can even make the computer easier to understand.

#### 3.1.2. Spatiotemporal Querying Agent

Consider the sensor locations that were included in Figure 7. If spatiotemporal information were encoded as RDF, how could it be ingested and indexed in such a way as to efficiently return the closest monitoring site to a particular location? Given a set of geospatial information in RDF, all of which are points, a system is created that is capable of efficiently answering the query, “Return all of the points that are within a distance X of a given location.”

Notions of space and time are intimately related to every sensor in the world, because every sensing event happens somewhere and some when. Almost every sensor network is concerned with the spatiotemporal aspect of data to some degree, though some deal only with the temporal plane. One of our goals is to develop appropriate RDF and OWL representations of spatiotemporal information from heterogeneous sensor networks. Any system that maintains information that can change must at some level deal with time; for instance, and many questions include some assumptions of location as well. For some sensor network applications, the connections to space and time are very obvious (e.g., the Radio-frequency identification (RFID) tag tracking applications). However, different sorts of sensor network applications use space or time more implicitly; more so, to contextualize an answer to a related query. For example, for a query asking about whether someone should do their morning exercise outside today, the answer is not exclusively dependent on the spatiotemporal sensor data, but the information from the nearby air quality monitoring sites must be considered. Being able to bound queries into a particular spatiotemporal region can be very useful.

This section presents the spatiotemporal development for the Query Agent. These challenges include not only abstract problems of information computing, but also practical concerns of program efficiency. A geospatial index in the agent, which is a special data structure, can be integrated into a Jena model, so as to make some of the most common types of spatial queries efficient.

**Figure 8:**
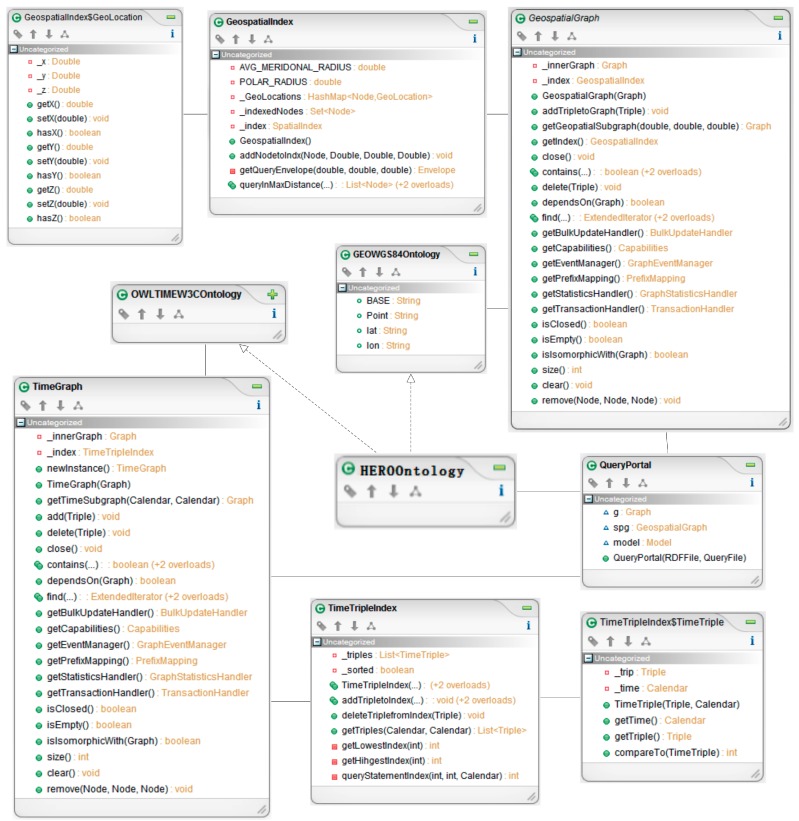
Spatiotemporal Query Agent Class Diagram.

The SpatiotemporalQuery Agent uses RDF serializations (RDF/XML, Turtle, or N-Triples format) to answer spatiotemporal sensor information queries. As Figure 8 shows, the GeospatialIndex class and TimeTripleIndex class wrap JTS Quadtree objects so that they can be easily used from Jena API, serving as the bridge between the program and JTS. The GeospatialGraph class and the TimeGraph class implement Jena’s Graph interfaces, and they are used to integrate the geospatial and temporal indexings into the higher-level Jena constructs. The QueryPortal class is used to read the RDF serializations and query strings. An inner class GeoLocation is used to represent the latitude, longitude, and elevation values, while the TimeTriple class represents the standard time.

There are two main methods in the GeospatialIndex class. The addNodetoIndex method adds a sensor node, represented as a RDF resource, and defined with a latitude and longitude to the underlying geospatial index. The queryInMaxDistance method creates a bounding box based on the user-defined distance, and uses the geospatial index to return all sensor nodes within the box.

The GeospatialGraph class wraps an existing Jena Graph object, and it has two methods of significance. The addTripletoGraph method inspects its parameter to determine whether it is a statement that describes a latitude or longitude value. If it is, the GeospatialGraph calls the addNodetoIndex method on its GeospatialIndex object to register the value. The getGeospatialSubgraph method is used to pose the geospatial queries of the model. Calling that method with latitude, longitude, and distance values, returns another Graph object containing only statements about the sensor locations within the specified region. This class addresses the challenge that a statement expresses only a single datum, but the spatial index requires both latitude and longitude values before a point can be indexed. Since the existence of one statement is independent of all others, it is possible that there could be a set of statements with an incompletely defined point, or of only a latitude or longitude. A final confirmation of all of the returned candidates is used to populate a list of sensor nodes that are ultimately returned to the calling code.

The agent, however, is not specifically limited to enhancing Jena for spatial data. A similar approach is used to incorporate a temporal index by using the TimeTripleIndex class and the TimeGraph class, allowing for queries that are bounded in transaction time.

Ontology components in the agent store the HERO Location and Time ontologies, which are designed according to the standard GEO W3C Ontology and the OWL-time Ontology, thus making it easier to share and interchange spatiotemporal information.

### 3.2. Case Study: S-SOS Agent for Querying Air Quality and Health Implications

The monitoring functions of the sensors in the Hubei Province Monitoring Stations in China are classified as ambient air monitoring, surface water environment monitoring, soil environment monitoring, environmental noise monitoring, and ecology & biological environment monitoring. The air monitoring sensors work with instruments such as the NO-NO_2_-NO_x_ analyzer, the SO_2_ analyzer, etc. Some citizen science applications are also implemented through websites and smart phones.

Automatic air monitoring sites were deployed in 17 cities in Hubei Province for daily air quality assessment. Real-time data are published to public via web services, including SO_2_, NO_2_, PM_10_, CO, O_3_, and PM_2.5_ hour values. The AQI of the sites, the primary pollutants, the levels of health concern, health implications, and cautionary statements are also published through official websites, APIs, and cellphone applications. The data are updated on an hourly frequency. AQI equals the maximum value of the IAQIs. IAQI is the individual Air Quality Index, which can be calculated, as Table 2 shows.

**Figure 9:**
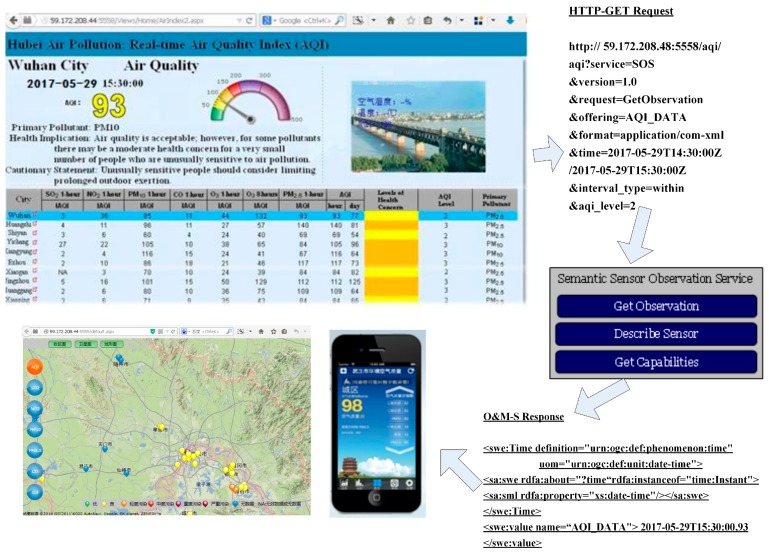
Prototypes of the S-SOS agent for publishing air quality.

We developed a prototype of the Semantic Sensor Observation Service (S-SOS) to add annotations to assist with obtaining health implications from air phenomena sensing. Semantically annotated air quality data are collected from the automatic monitoring sites in Hubei province, which is related to Location ontology, OWL-Time ontology, as well as HERO (see Figure 4). The rules between air quality and health implications, which Hubei province uses to publish data to the public, follow Table 3. According to the sensor data, the rules can deduce computational intelligence directly. Through observing the pollutants SO_2_, NO_2_, PM_10_, CO, O_3_, and PM_2.5_, we may know the “potential” air quality that AQI watched. The results and health suggestions will be published to the public automatically through official websites and smartphone APIs (see Figure 9).

**Figure 10:**
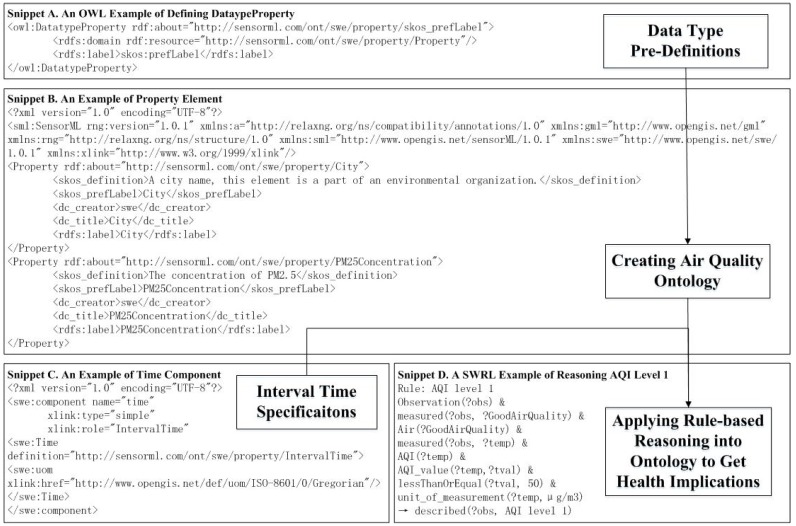
Processing an air quality and health implications querying Instance.

The ontology of a ground-sensing ambient air monitoring system is modeled for the case study. Figure 10 illustrates the procedures of applying rule-based reasoning into the ontology to obtain health implications. Snippet A of the OWL definition codes, mingled with RDF and RDFS, presents the properties of the referred data types. Snippet B of SensorML codes initializes the ontology of the ambient air quality. A skos:preLabel is created as the linked anchor, so that other components of the ontology can utilize this anchor to claim the properties for monitored cities and pollutant (like PM_2.5_) concentrations through the rdf:resource, http://sensorml.com/ont/swe/property. Since the ontology is initialized, services can be applied to manufacture reasonable ratiocination on the existing facts, and to derive new knowledge, and additional knowledge can be extracted by applying rule-based reasoning. Snippet C describes the specification of time, in order to illustrate that the PM_2.5_ concentrations are observed every hour. Snippet D is programmed as a rule-based reasoning example, based on an air quality model named WRF-EMEP (http://hubei-aq.info). Our reasoning proxy needs to figure out the AQI level automatically for the daily announcement. The pollutant concentrations can be converted to the IAQIs, of which AQI equals the maximum value. When the AQI is less or more than a certain threshold, the reasoning proxy deduces the new ontological assertion of the AQI level, which can guide health implications and cautionary statements. Through this method, referred websites or smart phone apps can forecast the risks to the nearby people in time.

### 3.3. Experiment and Evaluation

For evaluation purpose, we invited five health experts in Wuhan City, Hubei Province, to conduct field assessments. We sent them the health-related events and information near their locations for manual verification of the health events collected by the sensor networks. As a comparison, the S-SOS agent in the HaEHMS is using the sensor observation data to verify the same health events, determining whether to prolong outdoor excises in certain locations.

With the traditional approach, we usually obtain the observation data of the pollutants from certain monitoring sites that is nearest to the event location, and assess the AQI result of that location.

**Figure 11:**
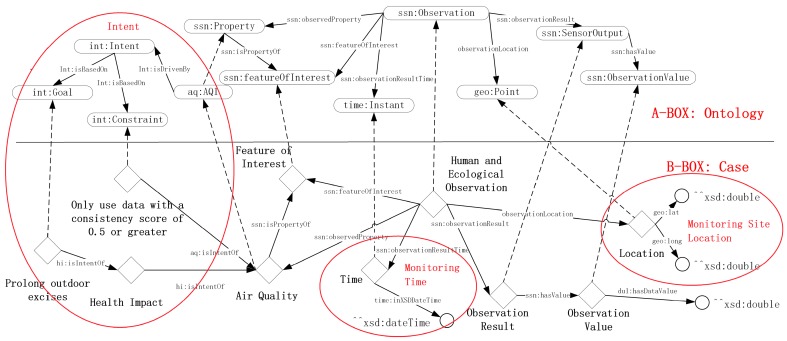
Ontology-case statements for querying air quality and health implications.

With the proposed methodology, as described in Section 3.1.2, the Spatiotemporal Querying Agent can run with the HERO to execute the query, “Return the case records of the monitoring sites that are within a distance to the expert’s location (for instance within a 10km radius).” Each record contained the sensor data of air quality at that site during the 20 days period. As shown in Figure 11, reasoning about air quality and health implications by using HERO allows a reasoner to infer “Location” and “Time“, as an assessment is performed. The ontology models in A-BOX work as the classes for representing the continuous monitoring objects at intervals from B-BOX. Furthermore, an Agent’s intent can also be captured (boxes with “int” namespace in Figure 11). In this example, the agent has a constraint that it can only use AQI value data with a minimum consistency score of 0.5.

In this example, the observation describes the AQI value of Wuhan city where the average of the high and low values in May is 93. If the observation has an AQI value of 23, it is annotated with a consistency score of 0.2473 (=23/93).

The evaluation was done in two stages:

(1) AQI calculation and inference through the HaEHMS: As described in Section 3.2, the resulted system outcome in each case record included AQI values, AQI levels, levels of health concern, health implications, and cautionary statements.

(2) Manual verification by the health experts in field: Results of the case record were sent to the experts. The experts manually collected the same set of information as collected by the sensors installed at this site and compared the outcomes of two different sets of information:

$$Accuracy=\frac{\sum \{Sensor\;outcome\;is\;identical\;to\;the\;expert\;field\;observation\}}{Total\;records\;at\;the\;site}$$

The accuracy is the ratio of the number of identical results (between the sensor system outcome and the expert field evaluation) over the total number of cases (the sensor observation records). The sensor system collected information is very close to the manual observation by the field experts (see Table 4).

We found differences in some columns. After a joint review by the researchers, it was found that the reasons were related to weather conditions. The fluctuation of weather conditions was an important interference factor, which caused the differences. However, for the cases without the weather condition interference, the inference outcomes of the HaEHMS and the experts were identical.

## 4. Discussion and Conclusions

As the sensor web has developed rapidly, people have begun to realize that there exists a lack of uniform operations for resource reallocation, resource sharing and standard representation for sensor data. A sensor data management structure should be developed to address the problem of a lack of mutual operability between sensor data. The OGC SWE norms make all kinds of sensors and sensor data attainable, searchable and workable for creators through the internet by clarifying service connectors that allow for the mutual and functional use of sensor data.

The contribution of this study focuses on the ontology-driven approach for integrating intelligence to manage human and ecological health risks in the GSW. Firstly, we proposed the SWMF on the basis of the SWE services, to conceal the heterogeneity of its bottom sensor networks, and to facilitate the communication between its corresponding particulars and all equipment parts. Thus, the SWMF makes application developments under its framework an easier job. The sustainable environmental sensing in this paper not only utilizes observation data from the natural environment, but also citizen science data from crowdsourcing applications. Environmental computing can also be interfaced with the SWMF. Encoding sensor and sensor networks illustrations and monitoring data with semantic web languages allows for more meaningful expression, superior visits, and the official analysis of sensor resources. Moreover, the HERO ontology that we designed based on the SSNO template, is not just for sustainable environmental sensing, but also provides a robust means of describing the processes including the methodology. Then, we developed a software agent for pervasive sustainable environmental sensing and computing through using HERO models. The S-SOS agent could offer various kinds of observation data, and more often, daily routine environmental monitoring data from different sites. The ontology reasoning in the S-SOS agent also plays the intent modeling role in deriving the data. Finally, we developed the HaEMHS, which is also an application and prototype based on the SWMF to integrate the existing monitoring data, models and processes in the context of environmental risk monitoring. In our prototype implementation, we used the Amazon cloud service architecture to solve the problem brought about by the complex calculation of big-data and models. The steps of automatically reasoning the air quality forecast are also illustrated.

In short, this work makes a vital step towards the creation of an exoteric, broad, mutually operable, and smart sensor web for public environmental supervising. Our future work will focus on: (1) the reliability of the result of the data processing and analysis over a multi-layer sensor web; (2) the integration of an architecture with an emergency response, which contains video on mobile devices running operating systems; and (3) addressing management issues, such as how to involve policy-makers, ‘stakeholders’, and other end-users, which are vital to the standard of corresponding decision-making matters and the evolution of useful interactive sustainable instruments.

## Figures and Tables

**Table 1:** Development environment and open sources for the implementation of the cloud service.

Development/Operation Environment	Opensource/Platform/IDE	URL
Cloud computing	Amazon EC2 (64bit)	http://aws.amazon.com/ec2/
Cloud storage	Amazon S3	http://aws.amazon.com/s3/
Operating	Amazon Linux AMI	http://aws.amazon.com/amazon-linux-ami/
Image processing algorithm	Orfeo Toolbox (OTB)	http://www.orfeo-toolbox.org/otb/
Image tiling and converting	Geospatial Data Abstraction Library (GDAL)	http://www.gdal.org/
Web server	Apache Httpd, Tomcat	http://tomcat.apache.org/
Programming language	Python,	https://www.python.org/
	JAVA	http://www.java.com/
Python interface to AWS	Boto	http://docs.pythonboto.org/en/latest/#
		https://github.com/boto/boto/
Parallel processing	Parallel Python	http://www.parallelpython.com/

**Table 2:** Conversion between pollutant concentrations and IAQI.

IAQI	SO_2_ (Averaged over 24 h)	SO_2_ (Averaged over 1 h)	NO_2_ (Averaged over 24 h)	NO_2_ (Averaged over 1 h)	PM_10_ (Averaged over 24 h)	CO (Averaged over 24 h)	CO (Averaged over 1 h)	O_3_ (Averaged over 1 h)	O_3_ (Averaged over 8 h)	PM_2.5_ (Averaged over 24 h)
50	50 ^1^	150	40	100	50	2	5	160	100	35
100	150	500	80	200	150	4	10	200	160	75
150	475	650	180	700	250	14	35	300	215	115
200	800	800	280	1200	350	24	60	400	265	150
300	1600	-	565	2340	420	36	90	800	800	250
400	2100	-	750	3090	500	48	120	1000	-	350
500	2620	-	940	3840	600	60	150	1200	-	500

^1^ Pollutant concentrations unit (μg/m^3^), where CO units (mg/m^3^).

**Table 3:** Air Quality and Health Implications.

AQI Values	AQI Levels	Levels of Health Concern	Colors	Health Implications	Cautionary Statements
0–50	1	Good	Green	Air quality is considered satisfactory, and air pollution poses little or no risk.	None
51–100	2	Moderate	Yellow	Air quality is acceptable; however, for some pollutants there may be a moderate health concern for a very small number of people who are unusually sensitive to air pollution.	Unusually sensitive people should consider limiting prolonged outdoor exertion.
101–150	3	Unhealthy for Sensitive Groups	Orange	Members of sensitive groups may experience health effects. The general public is not likely to be affected.	Active children and adults, and people with respiratory disease, such as asthma, should limit prolonged outdoor exertion.
151–200	4	Unhealthy	Red	Everyone may begin to experience health effects; members of sensitive groups may experience more serious health effects	Active children and adults, and people with respiratory disease, such as asthma, should avoid prolonged outdoor exertion; everyone else, especially children, should limit prolonged outdoor exertion.
201–300	5	Very Unhealthy	Purple	Health warnings of emergency conditions. The entire population is more likely to be affected.	Active children and adults, and people with respiratory disease, such as asthma, should avoid all outdoor exertion; everyone else, especially children, should limit outdoor exertion.
>300	6	Hazardous	Maroon	Health alert: everyone may experience more serious health effects	Everyone should avoid all outdoor exertion.

**Table 4:** Comparison of Different Verification Types.

Verification Type	Estimated Levels of Health Concern			
	Good (%)	Moderate (%)	Unhealthy for Sensitive Groups (%)	Unhealthy (%)
	Prolong Outdoor Excises	Limit Prolong Outdoor Excises	Limit Outdoor Excises	Avoid Outdoor Excises
Manual verification by health experts	87.5	90	95	100
